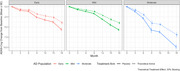# Breaking down the influences of placebo progression and percent slowing on the observed treatment difference in Alzheimer’s Disease clinical trials for populations in different disease stages

**DOI:** 10.1002/alz70859_103871

**Published:** 2025-12-26

**Authors:** Caleb W Dayley, Samuel P. Dickson, Kent Hendrix, Craig Mallinckrodt, Suzanne B. Hendrix

**Affiliations:** ^1^ Pentara Corporation, Salt Lake City, UT USA

## Abstract

**Background:**

Clinical trials aim to address three critical questions: the statistical significance of a treatment effect, the magnitude of that effect, and whether that effect is clinically meaningful. Symptomatic treatment effects can be assessed with actual point changes, or standardized changes (Cohen’s D) on clinical scales. However, in the context of progressive diseases like Alzheimer's Disease (AD), the percentage slowing approach can clarify the interpretation of treatment effects. This metric assesses the rate of disease progression observed in the placebo arm and the extent to which treatment slows that progression.

**Method:**

We extracted placebo progression data from early, mild, and moderate AD studies for ADAS‐Cog scores up to 18 months. Theoretical percent slowing effects were applied to the observed progression rates. This allowed us to evaluate the absolute and standardized treatment differences within each population for similar disease slowing, highlighting how the magnitude of these effects varies depending on disease stage.

**Result:**

Assuming an equal percent slowing across the populations, much larger point differences (170%) were seen in the moderate AD population compared to the early AD population (see Figure). The mild AD population demonstrates a slight increase in progression when compared to the early AD population. Despite these differences in progression, all three populations exhibit a comparable increase in the variance of change over the 18‐month observation period, meaning that the Cohen’s D values are dramatically different in these scenarios. The contrast of these results originates from the difference in disease progression between the different populations. As such, using percent slowing to evaluate treatment efficacy in the slow development phase provides a clearer picture of the true underlying story.

**Conclusion:**

Observed treatment effects must always be interpreted in the context of the specific population in which they are measured or they may be misinterpreted. Using rate of placebo progression to standardize a treatment effect allows evaluation of disease slowing effects in early disease. Without this change of perspective, we are likely to undervalue disease‐modifying treatment effects that may significantly improve the lives of patients in early stage, and overvalue symptomatic effects that are often observed in later stages.